# Efficient Particle Manipulation Using Contraction–Expansion Microchannels Embedded with Hook-Shaped Arrays

**DOI:** 10.3390/mi16010083

**Published:** 2025-01-13

**Authors:** Di Huang, Yan Zhao, Chao Cao, Jiyun Zhao

**Affiliations:** School of Mechanical and Electrical Engineering, China University of Mining and Technology, Xuzhou 221116, China; ts24050085a31@cumt.edu.cn (Y.Z.); caoch@cumt.edu.cn (C.C.)

**Keywords:** microfluidics, contraction–expansion array microchannel, hook-shaped arrays, inertial migration

## Abstract

Inertial microfluidics, as an efficient method for the manipulation of micro-/nanoparticles, has garnered significant attention due to its advantages of high throughput, structural simplicity, no need for external fields, and sheathless operation. Common structures include straight channels, contraction–expansion array (CEA) channels, spiral channels, and serpentine channels. In this study, we developed a CEA channel embedded with hook-shaped microstructures to modify the characteristics of vortices. Through experimental studies, we investigated the particles’ migration mechanisms within the proposed structure. The findings indicated that, in comparison to conventional rectangular microstructures, the particles within the hook-shaped microstructured CEA channels experienced a more pronounced influence from inertial lift forces. Moreover, the magnitude of the second flow within the novel configuration was directly proportional to the channel width, the length of the expansion segment, and the embedding depth of the microstructure. The innovative structure was subsequently employed for particle trapping, focusing, and separation. The experimental outcomes revealed focusing efficiency of up to 99.1% and sorting efficiency of up to 97%. This research holds the potential to enhance the foundational theory of Dean flows and broaden the application spectrum of inertial contraction–expansion microfluidic chips.

## 1. Introduction

Microfluidics technology facilitates the precise manipulation of microliter- and milliliter-scale samples through micron-scale flow channels. Owing to its superior capabilities in efficient particle manipulation, this technology has found extensive application in various biological processes, such as focusing [1], concentration [2], separation [3], and purification [4]. Leveraging these attributes, microfluidics technology enables the integration of sample injection, processing, and analytical characterization into a compact device, typically measuring only a few square centimeters, which is particularly advantageous for life science research. As a core technology, it plays a pivotal role in the development of point-of-care diagnostic devices and demonstrates substantial potential in addressing medical emergencies, advancing home-based diagnostics, and enhancing healthcare infrastructure in resource-constrained settings.

With the ongoing advancement of microelectronics technology, a variety of microfluidic chips have been developed, which can be categorized into active and passive types. Active manipulation technologies depend on external fields, such as electric [5], magnetic [6], acoustic [7], and optical [8] fields. While these methods provide high manipulation accuracy, they are characterized by low efficiency and necessitate the use of external field-generating equipment, resulting in increased costs, larger device dimensions, and more complex operations. Conversely, passive technologies, including micro-scale filtration [9], hydrodynamic [10,11], deterministic lateral displacement [12], and inertial [13] techniques, manipulate micro- and nanoparticles via specific channel designs or fluid dynamics. These methods typically offer notable benefits, such as high throughput, independence from external fields, and simplified chip designs, although their manipulation precision is comparatively lower.

Inertial microfluidics leverages the inertial effects of fluids at the micro-scale to attain precise control over particles’ migration and equilibrium positions. Common channel configurations encompass CEA channels, serpentine channels, and spiral channels. Notably, CEA channels, which represent deformations of straight channels, have garnered significant attention as a suitable tool for the manipulation of micro- and nano-scale biological particles in recent years. Sollier et al. [14] developed a technology that integrates micro-scale vortices and inertial focusing. The vortex chip employed in this study features eight parallel pathways, each consisting of a single straight high-aspect-ratio channel followed by eight rectangular reservoirs. Utilizing the vortex chip, CTCs were successfully isolated and quantified from the blood samples of breast and lung cancer patients with high purity. Liu et al. [15] introduced four CEA channels characterized by distinct aspect ratios and contraction–expansion angles, demonstrating that smaller expansion and contraction angles facilitated particle focusing. Notably, the circular channel exhibited the most effective focusing performance. This microfluidics system was successfully employed for the efficient and pure separation of plasma, red blood cells (RBCs), and cancer cells from blood. Yao et al. [16] introduced an innovative approach that combined contraction–expansion structures with three-dimensional sidewall electrodes, achieving the highly efficient sorting of lung cancer cells from red blood cells. Cha et al. [17] investigated the integration of periodic concave and convex obstacle microstructures into sinusoidal channels to further enhance the performance. Their findings demonstrated that concave obstacles notably strengthened the Dean flow and adjusted the flow range for particle inertial focusing and separation. The device effectively processed both polystyrene beads and breast cancer cells spiked in blood, achieving remarkable separation efficiency. Li et al. [18] employed a stepped microchannel, comprising a low-aspect-ratio straight section and multiple expansion regions along the channel height, to investigate the horizontal and vertical focusing, orientation, rotational, and translational behaviors of *Euglena gracilis* as functions of the aspect ratio (AR) and channel Reynolds number. Utilizing inertial focusing and local secondary flows, they successfully directed *E. gracilis*, despite their varied shapes, to a single equilibrium position within a single focal stream. This technique has potential applications in on-chip flow cytometry. Fan et al. [19,20] reported the design of microchannels featuring a series of abruptly contracted and extensively expanded structures on one side, which facilitated the focusing and ordering of microparticles into a single particle train. The authors observed that a horizontal vortex formed downstream of the contracted sections, effectively preventing particles from approaching the channel walls. Conversely, in the extensively expanded regions, the streamlines are curved without generating vortices, which drives the particles towards the walls of these expanded structures. Zhao et al. [21] introduced an innovative particle separation technique through the development of a straight microchannel featuring arc-shaped groove arrays patterned on its upper surface. The experimental findings demonstrated that the efficacy of particle focusing exhibited relative insensitivity to variations in the flow rate. In our previous work [22], we conducted an in-depth review of recent advancements in inertial microfluidics. A diverse array of channel geometries, including straight channels, curved channels, and CEA channels, were comprehensively discussed for their applications in focusing, concentrating, isolating, and separating various bioparticles. The study emphasized that the CEA channel retains the advantages of straight channels, such as a simple structure and ease in achieving parallel integration. However, due to the limited distance between the equilibrium positions of different particles, the separation efficiency remains relatively low compared to serpentine and spiral channels.

Despite the considerable advancements achieved by researchers, existing CEA structures typically exhibit insufficient induced vortex intensity, leading to suboptimal particle manipulation efficiency. To mitigate this challenge, this paper proposes a novel CEA channel integrated with a hooked array structure. In contrast to conventional CEA channels, the hooked microstructure boasts smoother curvature and an expanded downstream area, thereby enhancing vortex formation and improving the particle manipulation efficiency. Utilizing the prepared device, the influence of three parameters—the expansion section length, microstructure embedding depth, and main channel width—on the inertial focusing behavior of particles was systematically characterized. Subsequently, the device was employed to conduct a range of applications, including efficient particle capture, focusing, and sorting. Compared to the existing representative research achievements regarding CEA channels [23,24,25,26], our device exhibits multifunctionality, a broader application spectrum, and excellent overall performance.

## 2. Basic Theory

### 2.1. Inertial Migration Theory

The phenomenon of inertial migration was first reported in 1961, when Segre and Silberberg [27,28] discovered that suspended particles in a circular tube migrate to form an annular ring at a distance of 0.6 times the tube radius from the center when the Reynolds number is finite. This phenomenon is also referred to as the tubular pinch effect. In a straight channel, the fluid flow exhibits a Poiseuille velocity distribution, which generates a shear-induced inertial lift force. This force, arising from the shear velocity gradient, drives the particles towards the channel walls and is defined as the shear-induced inertial lift. As the particles approach the wall under the action of the shear-induced inertial lift, the symmetric wake generated by particle rotation is disturbed by the presence of the wall. This disturbance creates a repulsive force that drives the particles away from the wall, which is defined as the wall-induced inertial lift [29]. Under the combined influence of these two forces, the particles reach an equilibrium position. The combined effect of the forces is termed the inertial lift force. Asomolov [30] derived the expression for the inertial lift force as

(1) $$F_{L}=C_{L}\rho U_{m}^{2}a_{p}^{2}/D_{h}^{2}$$

In this equation, $C_{L}$ is the dimensionless lift coefficient, ρ is the fluid density, $U_{m}$ is the maximum fluid velocity, $a_{p}$ is the particle diameter, and $D_{h}$ is the hydraulic diameter.

### 2.2. Secondary Flow

When a fluid flows through a curved channel, the imbalance in the radial pressure gradient and the influence of centrifugal forces cause the fluid in the central region of the channel to move outward. On the other hand, due to the confinement of the channel, the fluid near the outer wall is compressed and flows back along the upper and lower walls, thereby forming two symmetrical vortices in the channel cross-section. This phenomenon is known as secondary flow or Dean flow [31]. The inertial migration of particles in curved channels is predominantly governed by the synergistic effects of the inertial lift and Dean flow, facilitating the lateral displacement of the particles. In such channels, particles are subjected to both the previously mentioned inertial lift and the drag force generated by the Dean flow. The magnitude of the Dean drag force can be quantified using the Stokes drag force, as illustrated in the following equation:

(2) $$F_{D}=3\pi \mu a_{p}U_{D}$$

In this equation, $U_{D}$ is the secondary flow velocity. Ookawara et al. [32] described the calculation of the average secondary flow velocity as $U_{D}=1.8\times 10^{-4}{De}^{1.63}$. This equation is extensively utilized in applied research for estimation purposes.

Previous research has demonstrated that incorporating contraction–expansion structures into straight channels can generate a secondary flow. The competition between the secondary flow and inertial lift will determine the equilibrium position of a particle’s migration. The particles’ inertial migration positions vary accordingly, thereby enabling size-based particle focusing or separation.

## 3. Materials and Methods

### 3.1. Device Design and Fabrication

**Figure 1:**
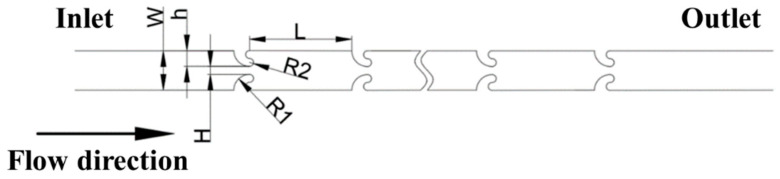
The scheme of the CEA microchannel embedded with hook-shaped arrays.

This paper introduces a microfluidic channel featuring embedded hooked microstructures. The microchannel is segmented into two primary components: the first component comprises a low-aspect-ratio rectangular channel, with a cross-sectional height of 60 µm and a width denoted as *W*; the second component consists of a periodically symmetric array of hooked microstructures positioned along both sides of the rectangular channel. Each hooked microstructure is constructed from a quarter-circle with a radius of *R*_1_ and two half-circles with a radius of *R*_2_, all of which are tangential to one another. The relationship between *R*_1_ and *R*_2_ is defined as *R*_2_ = 4*R*_1_. The minimum distance between the microstructures on either side of the main channel is *H*, the embedding depth of the hook-shaped microstructures is *h*, and the length of the expansion section is *L*. A total of 40 pairs of these symmetrically arranged hooked microstructures are present, forming a contraction–expansion microfluidic channel embedded with hooked microstructures, as illustrated in Figure 1.

The microfluidic device is fabricated utilizing a standardized soft lithography technique. First, a layer of negative photoresist (SU-8 2050, Microchem, Westborough, MA, USA) is deposited onto a silicon wafer via spin-coating, followed by the creation of a mold featuring protruding structures through ultraviolet (UV) exposure (MJB4, SUSS, Garching bei München, Germany) and subsequent development. Then, PDMS (a blend of a PDMS base and curing agent Sylgard 184, Dow Corning, mixed at a 10:1 ratio, Microchem, Westborough, MA, USA) is cast onto the mold, subjected to vacuum degassing, and thermally cured at 80 °C for a duration of 3 h. Upon the completion of the curing process, the PDMS sheet, now incorporating the channel structures, is carefully detached from the mold, and inlet and outlet ports are created using a precision puncher (Harris Uni-core, Sigma-Aldrich, St. Louis, MO, USA). Both the PDMS sheet and the glass substrate undergo surface activation in a plasma cleaner (PDC-002, Harrick Plasma, New York, NY, USA) for 3.5 min before being promptly aligned and bonded. To ensure optimal bonding strength, the assembled device is further subjected to thermal treatment in an oven at 120 °C for 3–4 h.

### 3.2. Experimental Setup and Sample Preparation

Prior to the experiment, the hooked CEA microchannel was initially cleansed with ethanol, a solvent with reduced surface energy, to prevent the formation of air bubbles within the PDMS channel. Subsequently, the channel was rinsed with deionized water to eliminate any remaining ethanol. Throughout the separation procedure, the hooked CEA microchannel apparatus was positioned on the platform of an inverted microscope (model STL-MF52, Guangzhou Micro-shot Technology Co., Ltd., Guangzhou, China), with the inlet and outlet being connected through two PTFE tubes to syringes containing the prepared polystyrene particle suspension and a sample collection vessel, respectively. A syringe pump (model LSP01-1A, Wenhao Co., Ltd., Suzhou, China) was employed to introduce the polystyrene particle samples into the CEA microchannel, with the flow rate regulated by modifying the pump settings. To evaluate the separation efficiency of the device, bright-field microscope images were acquired using a CCD camera (model STL-P23OIUA, Jiangsu Situoli Instrument Co., Ltd., Nanjing, China). Thereafter, 100 sequential images captured under identical conditions were compiled and analyzed using the ImageJ software (version 1.46r, NIH) to examine the statistical distribution of particle migration. To clearly visualize the particle trajectories, we selected “Standard Deviation” from the Projection Type options and applied this setting to the stacking strategy.

Experiments were carried out utilizing four distinct sizes of polystyrene microbeads, with diameters measuring 5 μm (cat. no. 6-1-0500, BaseLine Chromtech Research Centre, Tianjin, China), 7 μm (cat. no. 6-1-0700, BaseLine Chromtech Research Centre), 10 μm (cat. no. 6-1-1000, BaseLine Chromtech Research Centre), and 15 μm (cat. no. 6-1-1500, BaseLine Chromtech Research Centre). The particle suspensions were diluted to predetermined concentrations through the use of deionized water. Furthermore, a 0.5 wt% solution of Tween 20 (Sigma-Aldrich, St. Louis, MO, USA) was incorporated into the prepared suspensions to inhibit particle aggregation. Given the low concentration of polystyrene microspheres and the addition of a surfactant, the interparticle interactions could be approximated as negligible. To ensure the optimal dispersion of the particles, the suspension was subjected to ultrasonic treatment for ten minutes prior to each experiment. To quantitatively calculate the focusing efficiency and other relevant metrics, a hemocytometer was employed to determine the particle concentrations in both the inlet and outlet samples. All experiments involving quantitative analysis were conducted in triplicate, and the mean value was utilized to calibrate the analysis results.

## 4. Results and Discussion

### 4.1. Particle Migration Characterization

**Figure 2:**
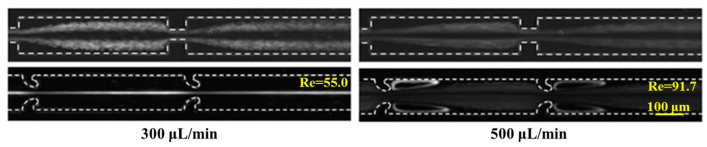
The inertial focusing behavior of particles in two types of CEA channels.

This study initially compares the migration behavior of particles within a hooked CEA channel against that within a standard CEA microchannel embedded with rectangular microstructures. As illustrated in Figure 2, at a flow rate of 300 µL/min, 7 µm particles are dispersed along the lateral edges of the channel in the conventional rectangular CEA channel, whereas, in the hooked CEA channel, the particles are entirely concentrated at the channel’s center. This difference is likely attributable to the predominance of secondary flows affecting particle movement in the conventional rectangular CEA channel, as opposed to the dominance of inertial lift forces in the hooked CEA microchannel. Upon increasing the flow rate to 500 µL/min, the focusing pattern of the 7 µm particles in the conventional contraction–expansion microchannel remains unchanged. Conversely, in the hook-shaped CEA channel, the particles are observed to become entrapped by vortices, suggesting that higher flow rates intensify the secondary flows in the hooked CEA channel more significantly than in the rectangular counterpart.

Given that the migratory characteristics of the particles are intricately linked to the channel’s structure, this study examines the influence of three structural parameters—the expansion segment length *L*, the embedding depth of the microstructure *h*, and the main channel width *W*—on the inertial migration behavior of the particles.

**Figure 3:**
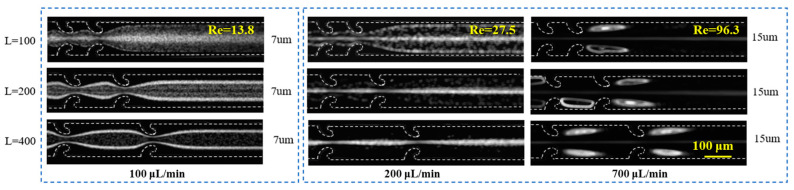
The impact of the expansion segment length *L* on the particle migration characteristics.

The experimental results regarding the impact of the expansion segment length *L* on the particle focusing characteristics are illustrated in Figure 3. It can be seen that, at a flow rate of 100 µL/min, when *L* is increased from 100 µm to 400 µm, the 7 µm particles progressively focus at two equilibrium positions near the channel walls. This observation suggests that, as the expansion segment length increases, the influence of secondary flows intensifies. In contrast, the 15 µm particles exhibit distinct focusing behavior. At a flow rate of 200 µL/min, as *L* increases, the 15 µm particles gradually accumulate in the channel center. At a higher flow rate of 700 µL/min, the 15 µm particles become entrapped by vortices, with the trapping effect becoming more pronounced as *L* increases. These findings indicate that, within a specific range, a longer expansion segment length facilitates the generation of stronger vortices and enhances the inertial effects within the hooked CEA channel.

**Figure 4:**
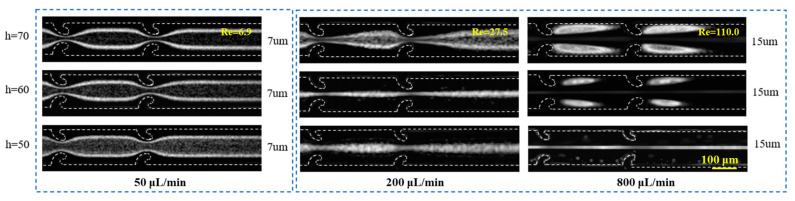
The impact of the embedding depth *h* on the particle migration characteristics.

The impact of the embedding depth *h* of the hook-shaped microstructures on the particle migration behavior was systematically investigated. As depicted in Figure 4, at a flow rate of 50 µL/min, when the embedding depth is 50 µm, the majority of the 7 µm particles are concentrated on both sides of the channel, and a significant number of particles remain dispersed in the central region. With an increase in *h*, the dispersion phenomenon progressively diminishes, and the 7 µm particles are almost entirely focused into two streams. At a flow rate of 200 µL/min, as *h* increases, the 15 µm particles exhibit a transition from random dispersion to central focusing and ultimately to lateral focusing. At a higher flow rate of 800 µL/min, when the embedding depth is 50 µm, the 15 µm particles are entirely focused at the center of the channel. However, as the embedding depth increases, the intensity of the vortices rises, resulting in increased particle trapping. The particle trapping rate is positively correlated with the embedding depth. These experiments highlight that increasing the embedding depth of the hook-shaped microstructures significantly enhances the strength of the secondary flows. By fine-tuning the embedding depth, the relative magnitudes of the drag force *F_D_* and lift force *F_L_* can be effectively controlled, thus manipulating the particle migration behavior.

**Figure 5:**
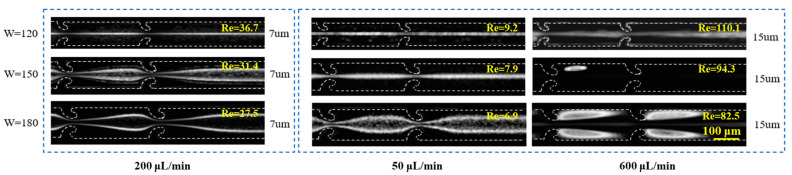
The impact of the main channel width *W* on the particle migration characteristics.

Finally, the impact of the main channel width *W* on particle migration was examined. As depicted in Figure 5, at a flow rate of 200 µL/min, as the main channel width expands from 120 µm to 180 µm, the 7 µm particles shift from being well focused at the center to being concentrated on both sides of the channel. This indicates the increased influence of secondary flows. Similarly, at a flow rate of 50 µL/min, the 15 µm particles display a comparable shift in behavior. At a higher flow rate of 600 µL/min, it is clear that a channel width of 180 µm generates the most pronounced secondary vortices, leading to a transition from central focusing (observed at a channel width of 120 µm) to vortex trapping (observed at a channel width of 180 µm) for the 15 µm particles. The aforementioned analysis indicates that the intensity of the secondary flows is directly proportional to the main channel width *W*. As *W* increases, the effect of secondary flows on the particles becomes markedly more pronounced.

In summary, the intensity of the secondary flows within the hook-shaped CEA microchannel is directly proportional to the length of the expansion segment *L*, the embedding depth of the microstructure *h*, and the width of the main channel *W*. By fine-tuning these structural parameters, specific particle behaviors, including center focusing, side focusing, and vortex trapping, can be selectively achieved. This finding highlights the broad spectrum of potential applications for our device.

### 4.2. Applications of Hooked CEA Channels

#### 4.2.1. Particle Trapping

**Figure 6:**
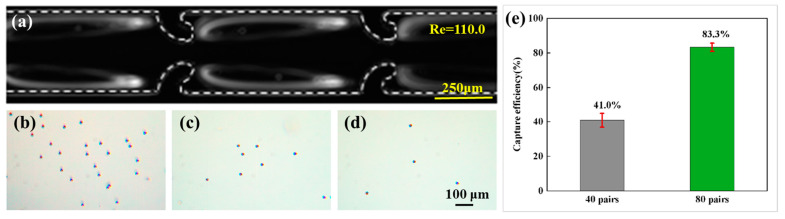
Particle trapping. (**a**) Particle migration trajectories. Microscopy images of the samples collected from the (**b**) inlet and (**c**) outlet of the CEA channel with 40 pairs of hook-shaped microstructures and (**d**) outlet of the CEA channel with 80 pairs of hook-shaped microstructures. (**e**) Quantitative analysis of the capture efficiency.

Based on the above research, particle trapping was conducted utilizing a hooked CEA channel with the specifications *W* = 180 μm, *L* = 400 μm, and *h* = 70 μm, and 40 pairs of hook-shaped microstructures. The flow rate was set to 800 μL/min. A syringe pump was employed to inject a suspension containing 15 μm particles with a concentration of $1.5\times 10^{5}$ particles/mL into the inlet of the channel. The particle migration trajectories within the channel are illustrated in Figure 6a. For the sample collection from the outlet (as shown in Figure 6c), a hemocytometer was utilized to quantify the particle concentration as $8.85\times 10^{4}$ particles/mL. The capture efficiency was defined as the ratio of the difference between the particle numbers at the inlet and outlet to the particle number at the inlet. Thus, it was found that the capture efficiency was 41.0%. The relatively low capture rate was primarily attributed to the limited capacity of the vortices. To enhance the capture efficiency, the number of hook-shaped microstructures was increased to 80 pairs, while all other experimental conditions remained constant. A microscopic image of the sample collection from the outlet is presented in Figure 6d, with a measured particle concentration of $2.5\times 10^{4}$ particles/mL, leading to the calculated capture efficiency of 83.3%. It should be noted that the aforementioned results are derived from samples collected between 20 and 40 s post-experiment initiation. As the experimental duration was extended, the occupancy rate of particles within the vortex increased, which could lead to a significant reduction in the capture efficiency. Furthermore, increasing the number of hook-shaped microstructures can further improve the trap efficiency. The performance suggest that our device is particularly suitable for capturing low-target-concentration samples. In addition, the trapping application has the potential to be extended to filtration, wherein particles of a specific size can be selectively trapped within the vortices, while particles of other sizes are directed towards the outlet.

#### 4.2.2. Particle Focusing

**Figure 7:**
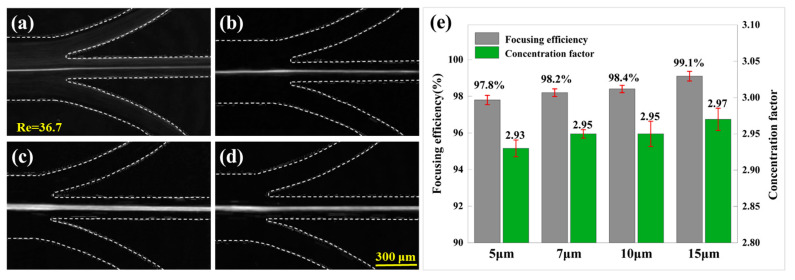
Particle focusing and concentration. Stacked composite images illustrating the distributions of (**a**) 5 μm, (**b**) 7 μm, (**c**) 10 μm, and (**d**) 15 μm particles at the outlets. (**e**) Quantitative analysis of the focusing efficiency.

To perform focusing and concentration experiments on particles with diameters of 5 μm, 7 μm, 10 μm, and 15 μm, a hook-shaped CEA channel characterized by *W* = 120 μm, *L* = 500 μm, and *h* = 40 μm was utilized. The microchannel outlet was configured with three equally divided branches, and the flow rate was set to 200 μL/min. Following passage through the hooked CEA channel, the distributions of the four types of particles at the outlet were as depicted in Figure 7, demonstrating effective focusing across all sizes. To quantitatively assess the focusing performance, the focusing efficiency was defined as the ratio of the number of particles in the middle outlet to the number of particles at the inlet, and the concentration factor was defined as the ratio of the particle concentration at the middle outlet to that at the inlet. The calculations revealed that the focusing efficiencies for particles with diameters of 5 μm, 7 μm, 10 μm, and 15 μm were 97.8%, 98.2%, 98.4%, and 99.1%, respectively, while the concentration factors were 2.93, 2.95, 2.95, and 2.97, respectively. These findings suggest that the device exhibits superior particle focusing capabilities across a broad range of particle sizes. The focusing performance is enhanced with increasing particle sizes, aligning with the principle that inertial forces are proportional to the particle diameter (or its square), as outlined in Equations (1) and (2). Furthermore, narrowing the width of the middle outlet can significantly improve the concentration factor.

#### 4.2.3. Particle Separation

**Figure 8:**
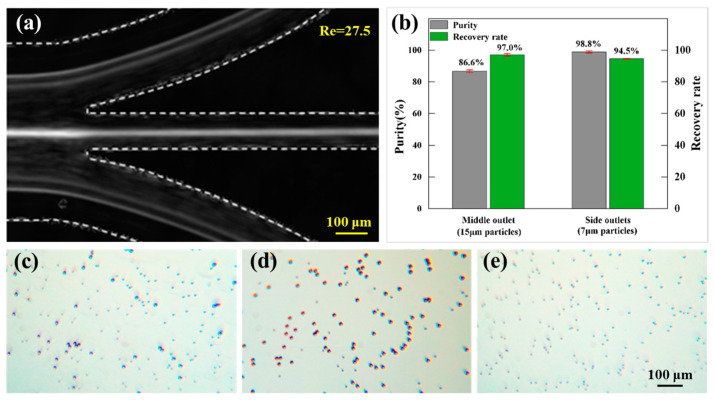
Particle separation. (**a**) Stacked composite images illustrating the 7 μm and 15 μm particle distributions at the outlets of the hooked CEA channel. (**b**) Quantitative analysis of the separation efficiency. Microscopy images of the collected samples from the (**c**) inlet, (**d**) middle outlet, and (**e**) side outlets.

A hook-shaped CEA channel characterized by *W* = 180 μm, *L* = 500 μm, and *h* = 60 μm was utilized for size-based particle separation. At the end of the channel, a bifurcated outlet system (side outlet–middle outlet–side outlet = 2:1:2) was applied. A 1-milliliter mixed suspension containing 7 μm particles (1.1 × 10⁶ particles/mL) and 15 μm particles (4.0 × 10⁵ particles/mL) was prepared and introduced into the microchannel inlet at a flow rate of 200 μL/min. The particle trajectory distribution at the outlets is illustrated in Figure 8a. It can be seen that the larger 15 μm particles were focused at the center of the channel and exited through the middle outlet, whereas the smaller 7 μm particles were concentrated near the channel walls and exited through the two side outlets, thereby achieving effective separation. Microscopic images of the samples at the inlet and outlets are presented in Figure 8b–d. The image of the middle outlet collection indicates that the majority of the particles were the larger 15 μm particles, while the side outlet collection predominantly contained the smaller 7 μm particles. We defined the recovery rate as the ratio of the target particle number at the outlet to that at the inlet and the purity as the ratio of the target particle number to the total number at the outlet. Based on the particle counts in the collections at each outlet, the recovery rate of 7 μm particles in the side outlets was calculated to be 94.5%, with purity of 98.8%. For the 15 μm particles in the middle outlet, the recovery rate was 97%, and the purity was 86.6%.

## 5. Conclusions

In this work, we innovatively developed a contraction–expansion microchannel embedded with hook-shaped arrays for the efficient manipulation of micron-scale particles. Firstly, we demonstrated that this novel design generates more pronounced secondary flows compared to conventional CEA channels equipped with rectangular microstructures. Secondly, we conducted a systematic investigation into the effects of the channel parameters on particles’ inertial migration. The results indicate that, within a specific range, the inertial forces increase with the length of the expansion section *L*, the embedding depth of the microstructures *h*, and the width of the main channel *W*. Lastly, based on these data, we explored the multifunctional applications of the hooked CEA channels, such as trapping through vortices, focusing, and the effective separation of 7 µm and 15 µm particles. The experimental results reveal trapping efficiency of up to 83.3%, size-insensitive focusing efficiency of 99.1%, and separation purity and recovery rates of 98.8% and 94.5%, respectively, demonstrating outstanding overall performance. Since our device manipulates particles based on their size, we believe that it is capable of trapping, focusing, and separating bio-particles such as blood components, circulating tumor cells, bacteria, microalgae, etc. The smooth curves within the hooked CEA channel facilitate a reduction in fluid shear forces, thereby better preserving the activity of biological particles and minimizing issues such as particle breakage, accumulation, and clogging. This research is expected to contribute to the development of fundamental theories of inertial effects, improve the efficiency of particle manipulation, and broaden the application spectrum of inertial microfluidic devices.

## Data Availability

The original contributions presented in the study are included in the article, further inquiries can be directed to the corresponding author.
